# Development and Evaluation of Spatio-Temporal Air Pollution Exposure Models and Their Combinations in the Greater London Area, UK

**DOI:** 10.3390/ijerph19095401

**Published:** 2022-04-28

**Authors:** Konstantina Dimakopoulou, Evangelia Samoli, Antonis Analitis, Joel Schwartz, Sean Beevers, Nutthida Kitwiroon, Andrew Beddows, Benjamin Barratt, Sophia Rodopoulou, Sofia Zafeiratou, John Gulliver, Klea Katsouyanni

**Affiliations:** 1 Department of Hygiene, Epidemiology and Medical Statistics, Medical School, National and Kapodistrian University of Athens, 115 27 Athens, Greece; kdimakop@med.uoa.gr (K.D.); esamoli@med.uoa.gr (E.S.); aanalit@med.uoa.gr (A.A.); srodopoyl@med.uoa.gr (S.R.); sofiazaf33@gmail.com (S.Z.); 2 Department of Environmental Health, Harvard TH Chan School of Public Health, Boston, MA 02115, USA; jschwrtz@hsph.harvard.edu; 3 Department of Epidemiology, Harvard TH Chan School of Public Health, Boston, MA 02115, USA; 4 MRC Centre for Environment and Health, Imperial College London, London SE1 9NH, UK; s.beevers@imperial.ac.uk (S.B.); nutthida.kitwiroon@kcl.ac.uk (N.K.); andrew.beddows@kcl.ac.uk (A.B.); b.barratt@imperial.ac.uk (B.B.); 5 National Institute for Health Research Health Protection Research Unit (NIHR HPRU) in Health Impact of Environmental Hazards, Imperial College London, London SW7 2AZ, UK; 6 Centre for Environmental Health and Sustainability, School of Geography, Geology and the Environment, University of Leicester, University Road, Leicester LE1 7RH, UK; j.gulliver@imperial.ac.uk

**Keywords:** air pollution, exposure modeling, land use regression, chemical transport models, machine learning, particulate matter

## Abstract

Land use regression (LUR) and dispersion/chemical transport models (D/CTMs) are frequently applied to predict exposure to air pollution concentrations at a fine scale for use in epidemiological studies. Moreover, the use of satellite aerosol optical depth data has been a key predictor especially for particulate matter pollution and when studying large populations. Within the STEAM project we present a hybrid spatio-temporal modeling framework by (a) incorporating predictions from dispersion modeling of nitrogen dioxide (NO_2_), ozone (O_3_) and particulate matter with an aerodynamic diameter equal or less than 10 μm (PM10) and less than 2.5 μm (PM2.5) into a spatio-temporal LUR model; and (b) combining the predictions LUR and dispersion modeling and additionally, only for PM2.5, from an ensemble machine learning approach using a generalized additive model (GAM). We used air pollution measurements from 2009 to 2013 from 62 fixed monitoring sites for O3, 115 for particles and up to 130 for NO_2_, obtained from the dense network in the Greater London Area, UK. We assessed all models following a 10-fold cross validation (10-fold CV) procedure. The hybrid models performed better compared to separate LUR models. Incorporation of the dispersion estimates in the LUR models as a predictor, improved the LUR model fit: CV-R^2^ increased to 0.76 from 0.71 for NO_2_, to 0.79 from 0.57 for PM10, to 0.81 to 0.66 for PM2.5 and to 0.75 from 0.62 for O_3_. The CV-R^2^ obtained from the hybrid GAM framework was also increased compared to separate LUR models (CV-R^2^ = 0.80 for NO_2_, 0.76 for PM10, 0.79 for PM2.5 and 0.75 for O_3_). Our study supports the combined use of different air pollution exposure assessment methods in a single modeling framework to improve the accuracy of spatio-temporal predictions for subsequent use in epidemiological studies.

## 1. Introduction

Epidemiological studies have been utilizing various air pollution exposure assessment methods to associate individualized exposure to air pollution concentrations and health effects [1,2,3]. Early studies [4,5,6] of long-term air pollution exposure assigned the concentration measurement from the nearest fixed monitoring site or applied interpolation methods (e.g., inverse distance weighing and kriging) to a geographical point of interest (usually at the participant’s residential address). A limitation of this approach is its limited ability to capture temporal variation in concentrations. More recently, land use regression (LUR) and dispersion/chemical transport models (D/CTMs) are among the most common methods applied to predict air pollution concentrations at a fine spatial scale for use in epidemiological studies [1,2,7,8,9]. D/CTM models can capture temporal variation, but are limited by the accuracy of the emissions inventories. Moreover, satellite aerosol optical depth (AOD) data haves been frequently used for predicting fine particulate matter pollution [3,10], especially in the lack of fixed site monitoring data and in the need of analysis of nation-wide/large population studies.

These more recent methods have been shown to be useful tools [11] for different epidemiological study designs since they can be extended to predict both the spatial and temporal variability of air pollution concentrations. For example, in time series and panel studies they can provide daily predictions, while in studies assessing long-term exposure health effects, the daily predictions obtained can be averaged over the time period of interest. In addition, these developments in exposure modeling provide spatially resolved daily estimates enabling an integrated assessment of health effects arising from both long-and-short-term exposures. However, they also have some limitations. LUR model development relies on air pollution measurements provided from a fixed monitoring network or is based upon specifically designed monitoring campaigns within a study [12]. As a result, a spatially sparse monitoring network or the limited temporal coverage of specifically designed monitoring campaigns may increase exposure measurement error. D/CTMs are based on the description of the physicochemical processes of air pollution, involving pollutant emissions sources and its precursors [13]. Therefore, they require high quality input data to produce accurate predictions. AOD data have contributed to developing national models with a spatial resolution of 1 × 1 km for particulate matter with an aerodynamic diameter equal or less than 2.5 μm (PM2.5) [14], but, as they measure PM within a height of several kilometers above ground, modelling is required to estimate concentrations at the height of the human breathing zone [10,11]. Additionally, values are missing on days with cloud coverage, which may be a significant problem for certain geographical areas and seasons. Therefore, models based only on AOD data may have increased uncertainty and may not allow the adequate assessment of intra-city variations.

Air pollution is a major environmental risk factor for human health [15,16] and it is crucial to provide epidemiological studies with accurate estimates of exposure. To overcome the limitations of single exposure assessment methods and to improve the accuracy of predictions, recent studies are combining methodologies or outputs from different methods. Incorporation of predictions from D/CTMs and/or satellite-derived air pollutant concentrations as predictor variables within a LUR model have been shown to improve the model performance in terms of predicting the spatial variability of air pollution [17,18]. On the other hand, very few studies have developed hybrid models by incorporating the output of different exposure assessment methods into a single spatio-temporal (ST) modeling framework [18,19,20].

Within the “Comparative evaluation of **S**patio-**T**emporal **E**xposure **A**ssessment **M**ethods for estimating health effects of air pollution” (STEAM) project we developed two hybrid ST modeling approaches for air pollutants, by combining LUR, dispersion and machine learning modeling, in the Greater London Area for the years 2009–2013. In the first approach, we incorporate the predictions from dispersion modeling of nitrogen dioxide NO_2_, ozone (O_3_; trioxygen) and particulate matter with an aerodynamic diameter equal or less than 10μm (PM10) and PM2.5 into a ST LUR model framework. In the second approach, we apply an ST-generalized additive model (GAM) combination of the predictions of individual models. For NO_2_, O_3_ and PM10 this is carried out by including the predictions of the two methods (LUR and CTM modeling), while for PM2.5 by including the predictions of three methods (LUR, CTM and an ensemble model using machine learning and models informed also by satellite data) as independent variables in the GAM.

## 2. Methods

### 2.1. Study Area

The Greater London study area has a total of 9,784,200 inhabitants (Census 2011 data; https://data.london.gov.uk/dataset/2011-census-demography, accessed on 1 March 2017) across 5373 census-based aggregation units, the Lower Layer Super Output Areas (LSOAs), of which the centroids (longitude, latitude) are located within the London Orbital Motorway (M25). Each LSOA within the study area has a minimum population of 1000 persons with an average population in 2010 of 1722 [21]. Within the same area there are 219,093 post codes which also have defined centroids which are population weighted. The Greater London Area has an exceptionally extensive air pollution monitoring network which allows testing the performance of various models as will be described in the following sections.

### 2.2. Air Pollution Monitoring Data and Enhanced PM2.5 Database

We obtained daily measured concentrations of NO_2_, O_3_, PM10 and PM2.5 for 2009 through 2013 from the London Air Quality Network [22], the Air Quality England [23] and from the Automatic Urban and Rural Network [24]. For NO_2_, PM10 and PM2.5 we formulated a database with the 24 h average measured pollutant concentration (μg/m^3^), while for O_3_ we calculated the daily maximum 8 h (8 h max) average concentration (μg/m^3^). NO_2_ measurements were available from 130 monitoring sites, PM10 from 115 sites and PM2.5 from 33 sites. In order to enhance the representation of PM2.5 monitoring sites in the study area, we combined a regression model and a random forest (RF) approach to predict PM2.5 concentrations at fixed sites with PM10 measurements (but without PM2.5 measurements). More details on the methods applied for PM2.5 can be found in [25]. This procedure was essential for informing the ST LUR and machine learning PM2.5 modeling development with a sufficient number of monitoring locations (n = 104). For O_3_, measurements from 62 sites were used located in an extended area including 9688 LSOAs. O_3_ is a secondary pollutant whose levels are mostly higher in rural areas than in urban settings. Therefore, we extended our study area to account for its formation properties and transport patterns.

**Figure 1:**
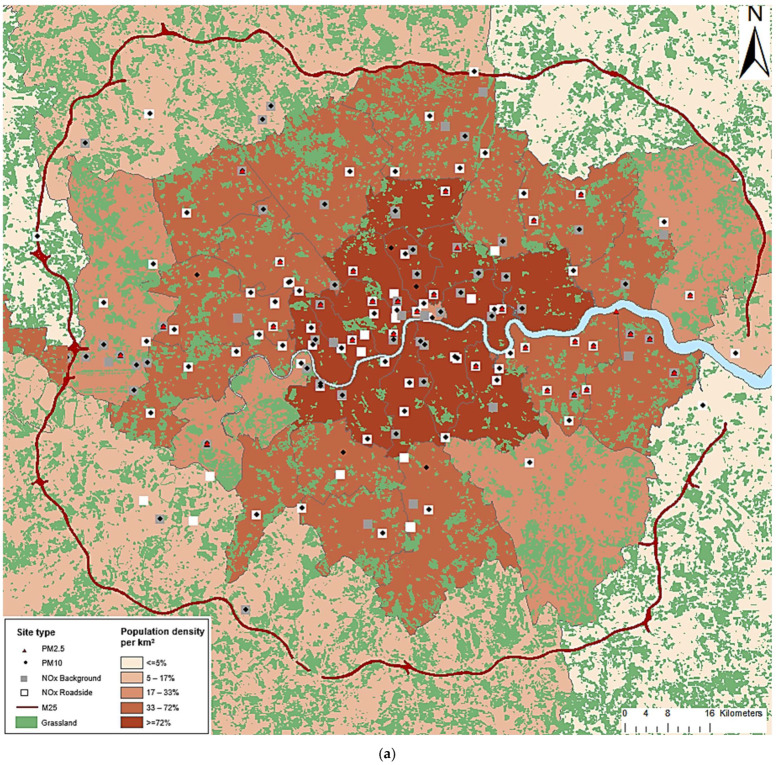

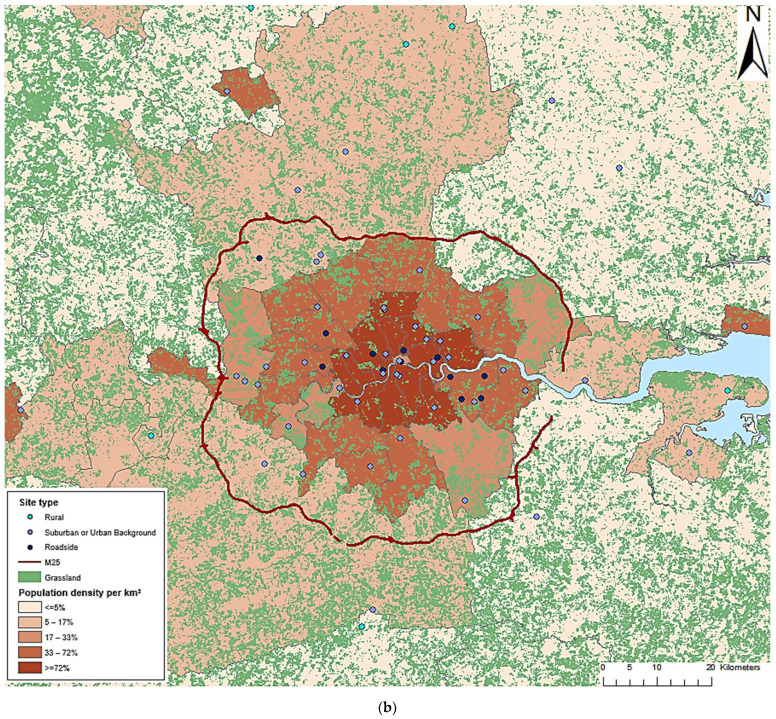
Map of the geographical location of the fixed monitoring network operated at the Greater London (**a**) study area for NO_2_, PM10 and PM2.5 and (**b**) extended study area for O_3_.

Figure 1 shows the study area and the geographical location of the monitoring sites.

### 2.3. Independent Exposure Assessment Methods

#### 2.3.1. Brief Summary of the First Stage Exposure Assessment

At the first stage ST LUR, dispersion and additionally machine learning models for PM2.5 were developed. The outputs of these approaches were combined under different hybrid model developed as the second stage. “Temporal” refers to daily (24 h) variation, while “spatial” refers to variation within the study area at the coordinates of interest (LSOA centroid).

#### 2.3.2. LUR Models

**Figure 2:**
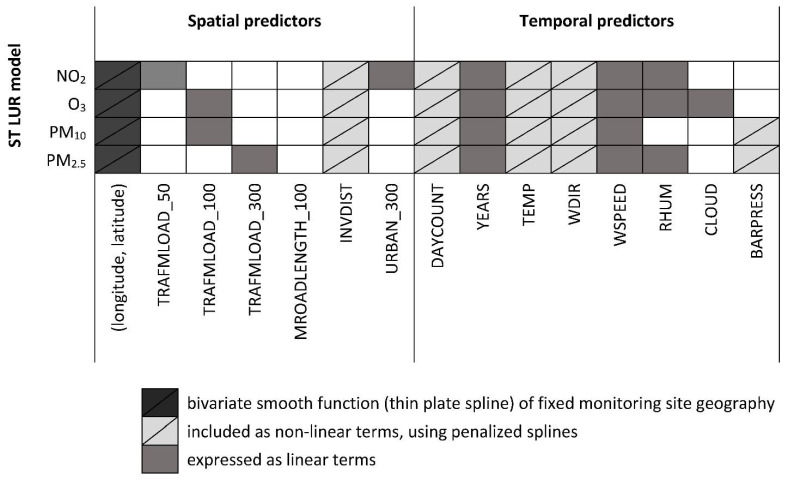
Predictor variables included in the final spatio-temporal land use regression (ST LUR) models developed for NO_2_, O_3_, PM10 and PM2.5 (μg/m^3^), in the Greater London Area for the years 2009 to 2013. TRAFMLOAD_50; TRAFMLOAD_100; TRAFMLOAD_300: traffic load of major roads (veh*m/day) in a buffer of 50m, 100m and 300m around each fixed monitoring site, respectively; MROADLENGTH_100: total length of major roads (m) in a buffer of 100m around each fixed monitoring site; INVDIST: inverse distance of fixed monitoring sites to the nearest major road (m^−1^); URBAN_300: urban areas (m^2^) in a buffer of 300m around each fixed monitoring site; DAYCOUNT: day count variable accounting for trends within each year coded from 1 to 365 or 366 (included penalized splines with 6 degrees of freedom (df)); YEARS: years of the study period (4 dummy variables with year 2009 as reference category); TEMP: daily mean temperature (°C, included penalized splines with 3 df); WDIR: daily mean wind direction (°N, included penalized splines with 3 df); WSPEED: daily mean wind speed (m/s); RHUM: daily mean relative humidity (%); CLOUD: daily mean cloud coverage (okta); BARPRESS: daily mean barometric pressure (mBar/hPa, included penalized splines with 3 df).

We developed semi-parametric ST LUR models to predict daily NO_2_, O_3_, PM10 and PM2.5 concentrations at any point of interest in the study area, for the years 2009 to 2013. Similar to the approach previously applied previously in Athens, Greece [26,27], the log-transformed air pollutant measurements (except for O_3_ as it was normally distributed) at location 𝑖 on day 𝑡 was modeled using a set of covariates that had either a linear or a smooth effect on the pollutant; plus a bivariate smooth function of the fixed monitoring sites’ geographical location (longitude, latitude) in order to account for remaining spatial correlation. We used available air pollution measurement data, while 97 potential predictors of air pollutants’ spatio-temporal variability were tested. These variables can be classified in four categories: (a) land use type (Land Cover Map—LCM of Great Britain from 2007) in a buffer range of 100 to 5000 m (m) around each fixed monitoring site (precisely, 100, 300, 500, 1000, and 5000 m); (b) meteorological variables, obtained from the UK Meteorological Office (daily mean): temperature (°C), relative humidity (%), wind direction (°N), wind speed (m/s), cloud coverage (okta), barometric pressure (mBar/hPa) and solar radiation (W/m^2^); (c) traffic-related: total length of major roads (m) in buffers of 25, 50, 100, 300, 500 and 1000 m, inverse distance of the fixed monitoring sites to the nearest major road (m^−1^), and traffic intensity on the nearest major road (veh day^−1^) to the fixed monitoring site and total traffic load within each buffer of 25 to 1000 m (veh day^−1^ *m). Traffic counts were obtained from the Department of Transport in the United Kingdom; (d) indicators: of linear trend within a year (a day count variable accounting for trends within each year coded from 1 to 365 or 366), of linear trend over the years of the study period (4 dummy variables with year 2009 as reference category) and the day of the week (6 dummy variables with Sunday as the reference category). For the smooth function we used a penalized spline with degrees of freedom (df) estimated via Restricted Maximum Likelihood (REML). Regarding all continuous variables, the final model included the term (linear or smoothed) that provided the better model fit. The final set of explanatory variables was selected based on the model’s adjusted-R^2^ value. Apart from the adjusted-R^2^ value, the coefficient of spatial covariates had to conform to the pre-defined direction of effect. The addition of variables was continued until none added more than 1% to the value of adjusted-R^2^. The final variables included in each of the ST LUR models are shown in Figure 2. All the predictor variables of the spatial variation of O_3_, PM10 and PM2.5 concentrations are traffic related. For NO_2_, the spatial variables included in addition to traffic-related variables, also a land use type (area characterized as urban within a buffer of 300 m) variable.

Regarding temporal variables, all final ST LUR models included meteorological and indicator variables for study years. Moreover, all final models included the geography (longitude, latitude) of the fixed monitoring sites. We validated our developed models using ten-fold cross-validation (10-fold CV). All land use types and traffic-related variables were extracted by conducting GIS analysis via ArcGIS Desktop, Release 10 [28]. All statistical analysis was conducted using the R statistical software (version 3.3.3; R Core Team, 2017, sourced from Athens, Greece) [29] and the R library “SemiPar” version 1.0-2 [30].

#### 2.3.3. Dispersion Models

We used a modeling system that combines the anthropogenic and natural emissions data, with the Weather Researching and Forecasting (WRF) meteorological model [31] and the Community Multiscale Air Quality (CMAQ) model [32], which has been coupled to the Atmospheric Dispersion Modelling System (ADMS) roads model [33,34] to predict hourly NO_2_, O_3_, PM10 and PM2.5 spatially at a 20 m grid resolution over the study area, for the time period 2009 to 2013. The anthropogenic emissions data were obtained by combining the UK National Atmospheric Emissions Inventory (NAEI) [35], the London Atmospheric Emissions Inventory [36], King’s road transport emissions model [37] and EMEP European emissions (https://www.ceip.at/, accessed on 15 June 2017). The biogenic emissions from vegetation and soils were estimated using the Biogenic Emission Inventory System version 3 (BEIS3) model [38]. Sea-salt emissions were calculated in-line in CMAQ. Bias in the 2 × 2 km CMAQ PM2.5 and PM10 hourly output was corrected using a sample of background sites before the local scale dispersion modelling stage. The discrepancies between the model output and the measurements at a random sample of 50% of background sites in the case of PM10, and 5 sites in the case of PM2.5, was interpolated onto the 2 × 2 km grid to create a correction surface. This interpolation was carried out using two iterations of a multilevel B-spline algorithm [39], which normally takes around eight iterations to interpolate points exactly, so that the resultant error surface provided smoothly varying bias correction across the domain, rather than fixing the model output to the measurements. The results from CMAQ-urban model were evaluated at 152 fixed monitoring stations from the UK and London monitoring networks, using methods described in the UK Department of the Environment, Food and Rural Affairs (DEFRA) model evaluation protocol [40].

#### 2.3.4. PM2.5 Prediction Model Based on an Ensemble Machine Learning ST Approach

We applied an ensemble machine learning approach including AOD, land use and meteorological data in order to predict daily PM2.5 concentrations in the study area, on a 1km × 1km scale (consisting in a total of 3960 grid cells). Details on the prediction model development can be found in [41]. In brief, the machine learners used in the process were the gradient boosting machine (GBM), the random forest (RF) and the k-nearest neighbor (KNN). AOD data were provided by the MAIAC algorithm for Moderate Resolution Imaging Spectroradiometer (MODIS) instrument on the Aqua and Terra satellites [42]. Predictors of the spatial variation of PM2.5 were: population density (persons/km^2^), land use type (LCM 2007), distance to water (km), distance to Heathrow airport (m), normalized difference vegetation index (NDVI), traffic counts, average daily PM2.5 across the greater London area (μg/m^3^), light at night, elevation (m), distance to nearest major road (km), distance to nearest bus stop (km), average building height (m) and length of major road (km), number of bus stops and number of buildings, in the grid cell. The included meteorological covariates were: cloudiness (okta), barometric pressure (mBar/hPa), wind direction (°N), wind speed (m/s), dew point temperature (°C), temperature (°C) and inverse of the height of the planetary boundary layer (m^−1^). Additionally, variables on the temporal scale were included to account for seasonal variations of PM2.5 (sine of day of the year, cosine of day of the year and day of week) and to account for long-term trends (number of days from time of origin and year). Model training was based using a grid search to optimize the hyper-parameters for the algorithms, and by taking into account the mean square error (MSE) and cross-validated R^2^ values as selection criteria. Following, we obtained the final ensemble-averaged PM2.5 predictions from a GAM, with independent variables a smoothed function (using a penalized spline with degrees of freedom estimated via REML) of the predictions obtained from each machine learning methods and by including a bivariate smooth function of latitude and longitude. Ten-fold CV was applied to evaluate model performance.

#### 2.3.5. Agreement between First Stage (Independent) Exposure Assessment Methods

Lin’s concordance correlation coefficient was calculated as a measure of agreement, at a temporal and spatial level, between the independent exposure assessment methods [43]. Agreement at the spatial level was investigated by the comparison of annual means of estimates (from the 3 methods) for each Lower Layer Super Output Area (LSOA), centroid. Agreement at the temporal level was investigated by taking into account the daily estimates, over the whole study area. Moreover, Bland–Altman method was applied in order to evaluate the mean differences and to estimate an 95% agreement interval of the differences (LoA) between the independent exposure assessment methods (ST LUR models, Dispersion models and the ensemble machine learning approach [44].

### 2.4. Spatio-Temporal Air Pollutant Modeling and Validation within a Hybrid Framework

#### 2.4.1. Brief Summary of the Second Stage Exposure Assessment

At the second stage we combined estimates from the first-stage approaches either by incorporating the dispersion model output in the LUR or by combining pollutant-specific output in a GAM mode and applying a smooth term per first stage approach estimate.

#### 2.4.2. Incorporation of Predictions from Dispersion Models and from an Ensemble Machine Learning Approach in the Case of PM2.5 within the LUR Model

Depending on the availability of the independent exposure assessment approaches, we developed hybrid models by incorporating estimates derived from dispersion modeling and the prediction model for PM2.5 from an ensemble machine learning approach, of the following form:

(1) $$f_{p}(poll_{it})=W_{lt}^{T}\;\beta +\sum_{l=1}^{q}f_{l}\left(s_{l,it}\right)+h\left(geog_{ij}\right)+\sum_{k=1}^{n}g_{k}f_{l}\left(M_{l,it}\right)+\varepsilon_{it}$$

where 𝑝𝑜𝑙𝑙_𝑖𝑡_ is the air pollutant concentration measurement at location 𝑖 on day 𝑡, 𝑓_𝑙_ (.) *l = 1, 2, ..., q* is a smooth function reflecting the non-linear effect of covariate 𝑆_𝑙__,__𝑖𝑡_ on the pollutant’s concentration 𝑝𝑜𝑙𝑙_𝑖𝑡_, 𝑆_𝑙__,__𝑖𝑡_ stands for the *l*th smoothed covariate, *h* is a bivariate smooth function of the fixed monitoring sites geographical coordinates *geog_ij_ = (longitude_i_, latitude_j_)* that account for residual correlation between locations 𝑖 and 𝑗, *W_lt_* is the vector of covariates that have a linear effect on 𝑝𝑜𝑙𝑙_𝑖𝑡_ and β is the corresponding vector of regression coefficients. *M_it_* is a ST smooth function of the predictions from the *k =* dispersion model and the additional prediction model for PM2.5_,_ with coefficient *g*. For *g_k_* = 0 model (1) is equivalent to the non-integrated ST LUR model. The errors (ε_𝑖𝑡_) are assumed to be independent and normally distributed, with a mean value of zero and a constant variance $\left(\sigma_{\varepsilon }^{2}\right)$. For NO_2_, PM10 and PM2.5 the air pollutant concentration measurement was log-transformed. We did not log-transform the O_3_ measurements since they were normally distributed. Therefore, for NO_2_, PM10 and PM2.5: 𝑓_𝑝_(𝑝𝑜𝑙𝑙_𝑖𝑡_) = 𝑙𝑜𝑔(𝑝𝑜𝑙𝑙_𝑖𝑡_), while for O_3_: 𝑓_𝑝_(𝑝𝑜𝑙𝑙_𝑖𝑡_) = 𝑝𝑜𝑙𝑙_𝑖𝑡_. Based on the above, the specific models constructed were: A hybrid ST LUR framework by incorporating predicted concentrations of NO_2_, O_3_ and PM10 from dispersion modeling and by incorporating predicted concentrations of PM2.5 from the dispersion the ensemble machine learning approach, as covariates within the ST LUR. These models are thereafter referred to as “Hybrid 1” and “ensemble”, respectively.

#### 2.4.3. Combination of Predictions Derived from LUR, Dispersion and for PM2.5 also Based on an Ensemble Machine Learning Modeling within a GAM

Similarly, depending on the availability of independent exposure assessment approaches we constructed a GAM, by fitting a smooth function (penalized splines) of the predictions from each method (LUR, dispersion and the prediction model based on an ensemble machine learning approach). The predictions reflect the daily estimated air pollutant concentrations at the fixed monitoring sites located in the study area. Since both the LUR model and the prediction model based on the machine learning use the air pollutant measurements during the model development procedure (dependent variable), the 10-fold CV predictions obtained from each method were used in the GAM. Our GAM has the following form:

(2) $$\begin{array}{l} poll_{it}=s\left(LUR\;pred_{10fold\;CV}.poll_{it}\right)+s\left(dispersion\;pred.poll_{it}\right)+\\ +s\left(ML\;pred_{10fold\;CV}.poll_{it}\right)+\varepsilon_{ij} \end{array}$$

where 𝑝𝑜𝑙𝑙_𝑖𝑡_ is the air pollutant concentration measurement at the fixed monitoring site location 𝑖 on day 𝑡, *s* is a penalized splines with the degree of smoothness based on the generalized cross validation criterion—GCV), $LUR\;pred_{10fold\;CV}.poll_{it}$ are the 10-fold *CV* predictions of the air pollutant concentrations obtained from the ST *LUR* models, $dispersion\;pred.poll_{it}$ are the estimated air pollutant concentrations obtained from dispersion modeling and $ML\;pred_{10fold\;CV}.poll_{it}$ are the 10-fold *CV* predictions of the air pollutant (available only for PM2.5) concentrations obtained from the ensemble machine learning approach, on location 𝑖 on day 𝑡. The final output from the GAM framework is the weighted-average daily predictions of air pollutants. The weighing of single methods was carried out using the smoothed function which allows each method to vary along the concentration range of the pollutants in case one method performs better than another in a specific concentration range. The specific models constructed were: a hybrid GAM by combining predicted concentrations of NO_2_, O_3_, PM10 and PM2.5 from the *LUR*, dispersion and PM2.5 prediction model based on an ensemble machine learning approach—hereafter, “hybrid 2”.

#### 2.4.4. Validation

The hybrid 1 and 2 models’ performance was evaluated using a 10-fold *CV* method. In this method, all models were fitted to N-10% fixed monitoring sites and the predicted concentrations were compared with the measured (observed) concentrations at the left-out sites. This procedure was repeated 10 times. Finally, the overall level of fit (based on the model’s R^2^ value) between the predicted and observed concentrations, across all sites, was calculated as a measure of our hybrid models’ performance. Moreover, we separately investigated the temporal and spatial validity of the developed hybrid models. To assess each model’s temporal validity, we regressed the difference between the daily predicted and the mean annual predicted pollutant concentrations over the difference between measured pollutant concentrations and their mean annual levels. In order to assess spatial validity, we regressed the mean annual predicted concentrations of each pollutant over the mean annual measured concentrations at the fixed monitoring sites. The level of temporal and spatial fit was evaluated by obtaining the models’ 10-fold *CV* R^2^ value.

#### 2.4.5. Application

Subsequently, the hybrid models were used to predict daily concentrations of NO_2_, O_3_, PM10 and PM2.5 concentrations per LSOA, by averaging the predictions in all post code centroids located within the LSOA. Thus, for the 5373 LSOAs included in the Greater London study Area we predicted pollutant concentrations for 219,093 postcode centroids.

## 3. Results

Table 1 presents the distribution of the estimated NO_2_, O_3_, PM10 and PM2.5 concentrations predicted from the first-stage (independent) and the ensemble/hybrid exposure assessment methods. The data shown are long-term (years 2009 to 2013) predicted concentrations of pollutants per LSOA, after averaging the predictions in all post code centroids within the area of the LSOA. The range of the number of postcode centroids per LSOA was 1 to 1585, with a median value of 32 postcodes. The ST- LUR predicted pollutant concentrations are slightly underestimated compared to measurements from fixed sites. Similarly regarding NO_2_ and PM10 predictions obtained from dispersion modeling.

Table 2 summarizes the agreement between the independent exposure assessment methods. Regarding NO_2_, O_3_ and PM10 we assessed the spatial and temporal agreement by comparing the predictions obtained from LUR and dispersion models, while for PM2.5 we compared per 2 methods at a time. The spatial agreement between LUR and dispersion models is moderate. The temporal agreement is better than the spatial. The pollutant which displays the highest spatial agreement between exposure assessment methods is NO_2_. The highest temporal agreement was observed for PM2.5, between the dispersion and ensemble machine learning approach. However, the mean difference is larger for NO_2_ and O_3_ compared to PM.

Table 3 presents the performance of the independent and the hybrid modeling approaches for NO_2_, O_3_, PM10 and PM2.5 concentrations at the fixed air pollution monitoring sites. The ST LUR model for NO_2_ performed well (CV-R^2^: 0.71) with better ability to predict spatially than over time (i.e., for NO_2_ spatial R^2^: 0.67 and temporal R^2^: 0.33), while for O_3_ and particulate matter, the ST LUR model performed moderately well. The dispersion compared to the LUR model performed similarly regarding the prediction of nitrogen dioxides and ozone and better regarding the prediction of particulate matter. Both hybrid modeling approaches (hybrid model 1 and 2) outperformed the independent models and improved the accuracy of predictions for all pollutants in terms of RMSE. Incorporating the estimates derived from dispersion models and the ensemble machine learning approach (only for PM2.5) into the LUR model (hybrid model 1), resulted in an increase in the CV-R^2^ value by 5% to 22%. The combination of estimates derived from the separate exposure assessment methods within a GAM, increased the CV-R^2^ value by 9% to 19%. The largest improvement in terms of CV-R^2^ was for PM10.

The hybrid modeling approaches for NO_2_ showed better ability to predict spatially than temporally, while for ozone and particulate matter the predicted better temporally (Table 4).

**Figure 3:**
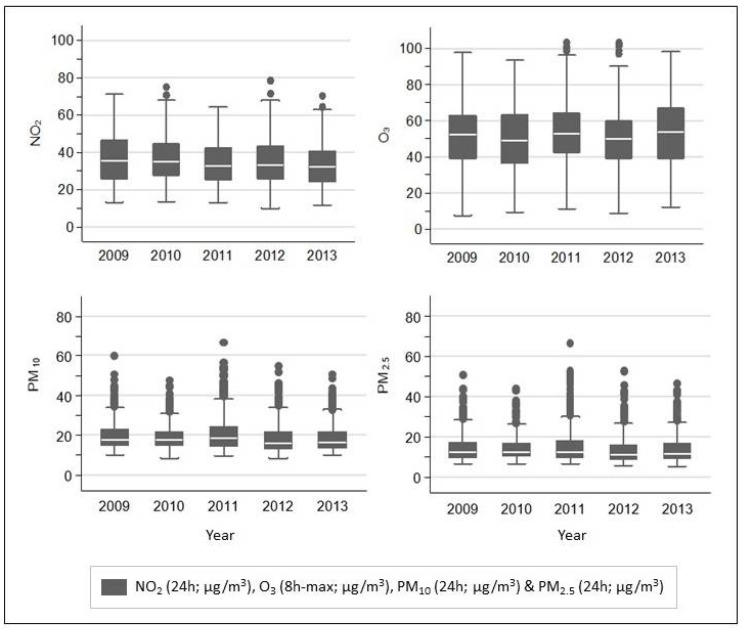
Yearly average (years 2009 to 2013) of estimated NO_2_ (24 h; μg/m^3^), O_3_ (8 h-max; μg/m^3^), PM10 (24 h; μg/m^3^) and PM2.5 (24 h; μg/m^3^) concentrations from the hybrid 2 model, in the Greater London Area. *Hybrid 2:* *Combination of estimates derived from independent exposure assessment methods, within a GAM*.

Figure 3 shows the yearly pattern (2009 to 2013) of the combined estimates derived from independent exposure assessment methods, within a GAM. The distribution of the estimated pollutant concentrations over the study period is similar. PM concentrations have more outliers compared to NO_2_ and O_3_ series.

**Figure 4:**
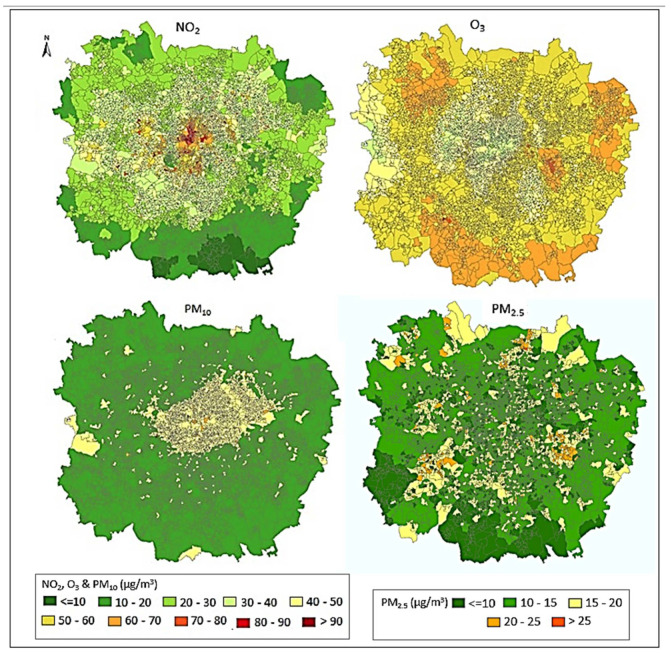
Long-term average (years 2009–2013) of estimated NO_2_ (24 h; μg/m^3^), O_3_ (8 h-max; μg/m^3^), PM10 (24 h; μg/m^3^) and PM2.5 (24 h; μg/m^3^) concentrations from the hybrid 2 model, per LSOA in the Greater London Area. *Hybrid 2:* *Combination of estimates derived from independent exposure assessment methods, within a GAM*.

Figure 4 displays an application of the combination of estimates derived from independent exposure assessment methods, within a GAM to predict pollutant long-term concentrations, per LSOA. The combination of estimates derived from ST LUR and dispersion models into a GAM framework (hybrid model 2) allows the relative weights for each model to vary spatially and by concentration and, therefore, display better performance.

## 4. Discussion

### 4.1. Findings

We developed a number of air pollution exposure assessment approaches for the Greater London Area, based on different methodological principles, to estimate concentrations in fine spatial (LSOA level) and temporal (daily) scales. For NO_2_, O_3_ and PM10 we developed a ST LUR and dispersion models, whilst for PM2.5 we additionally developed a model using machine learning algorithms and incorporating satellite data. These independent methods of pollutant concentration estimates are prone to errors from the uncertainty inherent to the measurement of variables used to develop the corresponding models. The errors are likely independent, as the dispersion modelling uses an emission inventory and information on atmospheric transformation processes influenced by the urban-scape, whilst the LUR models are based on air pollution measurements and the spatial and temporal variables determining their magnitude. So, it appears intuitively attractive to combine these methods, expecting that the errors of each separate method will cancel out. In the present project we used two types of combination models for each pollutant: one that incorporates the results of the dispersion model as a covariate in the LUR (hybrid 1) and one that combines the predictions from the 2 or 3 (for PM2.5) independent models with a GAM (Hybrid 2).

The Greater London Area has the advantage of a very dense monitoring network which provides measurements, including 130 sites for NO_2_, 115 for PM10, 62 for O_3_. The smaller number of sites measuring PM2.5 (n = 33) was enhanced by additionally using the data base developed in [25], resulting in the use of 104 sites. This network was used to predict the pollutant concentrations at each site and assess the exposure error and the agreement between methods.

Our results indicate that the combination models performed better in terms of cross-validated R^2^, RMSE and mean bias. It should be noted that for pollutants with high spatial variability and good knowledge on the determinants of this variability, such as NO_2_, models explain a larger proportion of this variability. For pollutants that are more spatially homogenous and tend to have larger temporal variability, the models tend to explain the temporal variability better. It is also interesting to note that the combination model 1 performs better for PM2.5 and PM10 whilst combination model 2 better for the gaseous pollutants.

### 4.2. Evaluation of the Combined Modeling Performance

Relatively few studies have compared the performance of model combinations of LUR and dispersion models at daily and fine spatial scales. The temporal scale ranges in the published work from annual to monthly or biweekly and seldom to daily. Additionally, the models have been developed for very different geographical areas and although the concepts are often similar, the models developed and those compared follow very different methodologies. Some studies compared different prediction methods (e.g., regression models and machine learning algorithms) using the same predictors and generally find that the application of machine learning methods yields better predictions [45,46,47]. 

De Hoogh et al. [18] compared the performance of LUR and dispersion modelling but did not assess the performance of any combination model. In a later work, De Hoogh et al. [48] developed and evaluated an extended LUR model for predicting annual concentrations in Western Europe for PM2.5, NO_2_, BC and O_3_, incorporating dispersion model estimates, kringing, satellite observations in addition to the LU variables. The combined full model performed better compared to less sophisticated models. This work was extended to eight elemental PM components [49]. Akita et al. [17] compared the performance of several models with a combined one using Bayesian Maximum Entropy in predicting annual NO_2_ concentrations in Catalunya, Spain, and report that their proposed combined framework outperformed the more conventional (LUR, Dispersion and others) approaches based on RMSE and other indices. Wang et al. [19] developed a combined LUR and chemical transport model, using a geostatistical modelling framework, for bi-weekly estimation of O_3_ and PM2.5 in the Los Angeles Basin and report that the combined model outperformed the initial models especially improving the accuracy of O_3_ predictions. Tripathy et al. [50] developed a combined model (“Hybrid”) for PM2.5, BC and metal components for the Pittsburg area, which performed better for PM2.5 than for the other components, but its performance was not compared to other models.

We compared the performance of models in terms of accuracy and bias in the predictions in space and time. Other papers evaluate how well the models perform in terms of providing valid and accurate estimates of the pollution exposure association with health outcomes. The optimal methods under these assessments do not necessarily coincide [51]. The exposure assessment methods presented in this paper have been evaluated in terms of their performance in estimating health effects using simulations that indicated the Hybrid 2 model to perform better both for PM and gaseous pollutants [52,53].

### 4.3. Advantages and Limitations

Our work has some advantages: it relies on a very dense and extensive monitoring network allowing many points in space for validation. Additionally, it combines exposure assessment approaches widely used in epidemiological studies. Further it assesses concentration estimates in a very fine spatial and temporal scale. However, it also has a number of limitations. London has a dense air pollution monitoring network. This may not be the case in other urban settings, especially those suffering from poor air quality. In such cities, the lack of measurements may limit the possible modelling and hybrid approaches. However, this could be overcome by designing a specific monitoring campaign or by applying methods to enhance existing measurement data bases [25]. The methods evaluated are only a subset of those that can be developed for the same area. Thus, other models may incorporate further data as available for example satellite data which were only used for PM2.5 in our study and other algorithms for prediction. Other types of combinations to produce hybrid models may be used. Models using land use/cover variables to predict air pollutant concentrations should be periodically updated to capture any changes in land use. In Europe, freely available land use databases (i.e., CORINE, Urban Atlas) are updated every 6 years. Data from local sources could be included to account for intense or fast changes in land use and therefore improve predictive performance of LUR models. Additionally, the transferability of the comparison results to other areas is questionable. As many characteristics determining space and time specific pollutant concentrations depend on the local topography, urban characteristics, population behavior and climate, our results may not be readily transferable to other locations and this aspect should be further investigated.

## 5. Conclusions

In conclusion, we show that combination or hybrid exposure models combining independent modelling methods based on different methodological principles perform better in terms of valid and accurate estimations of concentrations in time and space. This is broadly in accordance to the sparse and not directly comparable results that have already been published for other geographical locations. Future work should further evaluate methods that combine approaches (often termed “hybrid” to denote a variety of combinations) which appear consistently to outperform the single method approach.

## Figures and Tables

**Table 1:** Distribution of air pollutant concentrations measured from the fixed monitoring network and of estimated concentrations by the independent and hybrid exposure assessment methods, by pollutant, in the Greater London Area (n = 5373 LSOAs) for the years 2009–2013.

	Concentrations	Pollutant Measurements (μg/m^3^) Mean (SD) and *Median* (*25th–75th p)*			
		NO_2_ (24 h Average)	O_3_ (8 h Max)	PM10 (24 h Average)	PM2.5 (24 h Average)
**Fixed monitoring network**	**# sites**	130	62	115	104
		52.1 (24.39) *46.9* (*36.7–57.3)*	53.2 (11.62) *54.5* (*47.0–62.1)*	24.2 (5.18) *23.6* (*20.5–27.7)*	14.5 (2.48) *14.3* (*12.9–16.1)*
		**Estimations of Pollutant Concentrations (μg/m^3^)** **Mean (SD) and *Median* (*25th–75th p)***			
**Independent exposure assessment methods**	**LUR ^1^**	41.4 (20.77) *38.2* (*28.2–75.5)*	50.8 (8.23) *51.9* (*45.8–56.3)*	20.3 (4.01) *19.7* (*17.6–22.4)*	12.9 (2.12) *11.9* (*9.2–15.5)*
	**Dispersion ^2^**	37.7 (12.76) *35.8* (*29.4–43.0)*	59.8 (6.66) *60.9* (*56.0–64.9)*	19.7 (2.56) *19.1* (*17.9–20.9)*	14.2 (1.80) *13.8* (*12.9–15.3)*
	**Ensemble ^3^**	-		-	15.8 (1.28) *15.7* (*15.0–16.5)*
**Hybrid exposure assessment methods**	**Hybrid 1 ^4^**	43.8 (34.31) *35.1* (*25.5–48.7)*	57.7 (12.77) *54.1* (*49.2–58.8)*	21.9 (8.67) *20.0* (*17.5–23.6)*	14.1 (2.47) *13.7* (*12.3–15.3)*
	**Hybrid 2 ^5^**	35.2 (13.47) *33.6* (*26.7–41.5)*	51.7 (7.27) *52.6* (*47.2–56.8)*	19.5 (2.29) *18.9* (*17.9–20.5)*	14.4 (1.18) *14.2* (*13.6–15.1)*

^1^ spatio-temporal Land Use Regression (LUR) model. ^2^ spatio-temporal dispersion model. ^3^ PM2.5 prediction model based on an ensemble machine learning spatio-temporal approach. ^4^ Incorporation of estimates derived from independent exposure assessment methods, within the LUR model. ^5^ Combination of estimates derived from independent exposure assessment methods, within a GAM.

**Table 2:** Summary of agreement between the independent exposure assessment methods.

Agreement	Pollutant (μg/m^3^)					
	NO_2_ (24 h Average)		O_3_ (8 h Max)		PM10 (24 h Average)	
	Spatial	Temporal	Spatial	Temporal	Spatial	Temporal
Mean difference ^1^ (95% LoA)	−3.7 (−40.9, 33.4)	−3.7 (−18.6, 11.1)	9.0 (−6.7, 24.8)	9.0 (−18.5, 36.7)	−0.6 (−8.4, 7.1)	−0.6 (−12.0, 10.8)
Lin’s	0.39 *	0.78 *	0.25 *	0.61 *	0.31 *	0.69 *
r	0.45 *	0.82 *	0.44 *	0.71 *	0.37 *	0.75 *
	**PM2.5 (24 h Average; μg/m^3^)**					
	^a^ difference		^b^ difference		^c^ difference	
	Spatial	Temporal	Spatial	Temporal	Spatial	Temporal
Mean difference ^2^ (95% LoA)	0.4 (−4.4, 5.3)	0.4 (−4.5, 12.4)	−1.6 (−5.0, 1.8)	−1.6 (−5.2, 2.0)	−2.0 (−6.7, 2.7)	−2.0 (−11.9, 7.9)
Lin’s	0.26 *	0.67 *	0.24 *	0.96 *	0.12 *	0.71 *
r	0.28 *	0.77 *	0.39 *	0.98 *	0.22 *	0.81 *

Mean difference ^1^: Applicable to NO_2_, O_3_ and PM10; difference = Dipsersion—LUR predicted concentrations. Mean difference ^2^: Applicable only to PM2.5; ^a^ difference = Dispersion—LUR predicted concentrations; ^b^ difference = Dispersion—ensemble machine learning approach model predicted concentrations; ^c^ difference = LUR—ensemble machine learning approach model predicted concentrations. Lin: Lin’s concordance correlation coefficient. r: Pearson correlation coefficient. Ninety-five percent limits of agreement (LoA) Bland–Altman method. * *p*-value < 0.05.

**Table 3:** Model performance evaluated by the value of adjusted R^2^ and 10-fold cross validated (CV) R^2^, root mean square error (RMSE) and mean bias, for the independent and hybrid modeling approaches.

	Pollutant (μg/m^3^)			
	NO_2_ (24 h Average)	O_3_ (8 h Max)	PM10 (24 h Average)	PM2.5 (24 h Average)
**Number of Fixed Monitoring Sites**	130	62	115	104
**Independent exposure assessment methods**				
**ST LUR model ^1^**				
R^2^_adj_ and (CV-R^2^)	0.72 (0.71)	0.69 (0.62)	0.61 (0.57)	0.69 (0.66)
RMSE	4.28	13.67	7.42	3.64
Mean bias ^2^	−5.60	−0.14	0.96	0.59
**Dispersion ^3^**				
R^2^_adj_ and (CV-R^2^)	0.73 (0.70)	0.60 (0.59)	0.71 (0.69)	0.75 (0.74)
RMSE	4.13	15.60	6.41	4.26
Mean bias ^2^	0.73	−0.02	0.81	0.45
**Ensemble ^4^**				
R^2^_adj_ and (CV-R^2^)	-	-	-	0.88 (0.83)
RMSE	-	-	-	
Mean bias ^2^	-	-	-	0.058
**Hybrid exposure assessment methods**				
**Hybrid 1 ^5^**				
R^2^_adj_ and (CV-R^2^)	0.84 (0.76)	0.79 (0.75)	0.82 (0.79)	0.84 (0.81)
RMSE	3.71	10.24	2.72	0.20
Mean bias ^2^	−7.13	−11.12	−0.35	0.13
**Hybrid 2 ^6^**				
R^2^_adj_ and (CV-R^2^)	0.81 (0.80)	0.76 (0.75)	0.77 (0.76)	0.80 (0.79)
RMSE	3.64	11.94	4.02	1.91
Mean bias ^2^	1.64	0.03	0.68	0.36

^1^ developed spatio-temporal (ST) LUR models. RMSE: Root Mean Square Error. ^2^ bias = measured concentrations from fixed monitoring sites—10-fold CV predicted concentrations. ^3^ spatio-temporal dispersion model. ^4^ PM2.5 prediction model based on an ensemble machine learning spatio-temporal approach. ^5^ Incorporation of estimates derived from independent exposure assessment methods, within the LUR model. ^6^ Combination of estimates derived from independent exposure assessment methods, within a GAM R^2^_adj_: Adjusted R^2^ value of model. CV: 10-fold cross validation. CV -R^2^: R^2^ value of cross validated model.

**Table 4:** Temporal and spatial fit of the hybrid modeling approaches. Results from 10-fold cross validation.

	**Pollutant (μg/m^3^)**							
	**NO_2_** **(24 h Average)**		**NO_2_** **(24 h Average)**		**NO_2_** **(24 h Average)**		**NO_2_** **(24 h Average)**	
	**R^2^_-_** **Spatial**	**R^2^_-_** **Spatial**	**R^2^_-_** **Spatial**	**R^2^_-_** **Spatial**	**R^2^_-_** **Spatial**	**R^2^_-_** **Spatial**	**R^2^_-_** **Spatial**	**R^2^_-_** **Spatial**
**Hybrid 1 ^1^**	0.67	0.61	0.59	0.74	0.52	0.70	0.47	0.82
**Hybrid 2 ^2^**	0.72	0.63	0.61	0.72	0.62	0.76	0.59	0.87

^1^ Incorporation of estimates derived from independent exposure assessment methods, within the LUR model. ^2^ Combination of estimates derived from independent exposure assessment methods, within a GAM. R^2^: R^2^ value of 10-fold cross validated model.

## Data Availability

Publicly archived datasets analyzed in this study: Census 2011 data; https://data.london.gov.uk/dataset/2011-census-demography (accessed on 1 March 2017); London Air Quality Network (LAQN). King’s College, London. Available online: http://www.londonair.org.uk/ (accessed on 1 March 2017); London Datastore (accessed on 13 April 2020). Available online: https://data.london.gov.uk/dataset/lsoa-atlas (accessed on 18 November 2016); Air Quality England (AQE). Ricardo Energy and Environment. Available online: http://www.airqualityengland.co.uk/ Automatic Urban and Rural Network (AURN) (accessed on 1 March 2017). Data Archive: Available online: https://uk-air.defra.gov.uk (accessed on 6 December 2016); Land Cover Map. Available online: http://www.ukso.org/static-maps/land-cover-map.html (accessed on 29 November 2016); UK Meteorological Office, Available online: https://www.metoffice.gov.uk/research/climate/maps-and-data/historic-station-data (accessed on 21 November 2016); Department of Transport in the United Kingdom, Available online: https://data.gov.uk/dataset/d1f9e79f-d9db-44d0-b7b1-41c216fe5df6/national-public-transport-data-repository-nptdr (accessed on 4 August 2017); EMEP European emissions (accessed on 15 June 2016), Available online: https://www.ceip.at/; CERC, ADMS Roads v4 User Guide. (accessed on 15 December 2016) Available online: http://www.cerc.co.uk/environmental-software/assets/data/doc_userguides/CERC_ADMS-Roads4.0_User_Guide.pdf (accessed on 15 December 2016); Greater London Authority (GLA). The London Atmospheric Emissions Inventory 2013. Available online: https://data.london.gov.uk/dataset/london-atmospheric-emissions-inventory-2013 (accessed on 6 December 2016).

## References

[1] Beelen R., Raaschou-Nielsen O., Stafoggia M., Andersen Z.J., Weinmayr G., Hoffmann B., Wolf K., Samoli E., Fischer P., Nieuwenhuijsen M. (2014). Effects of long-term exposure to air pollution on natural-cause mortality: An analysis of 22 European cohorts within the multicentre ESCAPE project. Lancet.

[2] Cesaroni G., Badaloni C., Gariazzo C., Stafoggia M., Sozzi R., Davoli M., Forastiere F. (2013). Long-term exposure to urban air pollution and mortality in a cohort of more than a million adults in Rome. Environ. Health Perspect..

[3] Kloog I., Koutrakis P., Coull B.A., Lee H.J., Schwartz J. (2011). Assessing temporally and spatially resolved PM2.5 exposures for epidemiological studies using satellite aerosol optical depth measurements. Atmos. Environ..

[4] Jerrett M., Burnett R.T., Kanaroglou P.S., Eyles J., Brook J.R., Giovis C., Finkelstein N. (2001). A GIS—environmental justice analysis of particulate air pollution in Hamilton, Canada. Environ. Plan. A.

[5] Miller K.A., Siscovick D.S., Sheppard L., Shepherd K., Sullivan J.H., Anderson G.L., Kaufman J.D. (2007). Long-term exposure to air pollution and incidence of cardiovascular events in women. N. Engl. J. Med..

[6] Beelen R., Hoek G., van den Brandt P.A., Goldbohm R.A., Fischer P., Schouten L.J., Jerrett M., Hughes E., Armstrong B., Brunekreef B. (2008). Long-term effects of traffic-related air pollution on mortality in a Dutch cohort (NLCS-AIR study). Environ Health Perspect..

[7] Tonne C., Elbaz A., Beevers S., Singh-Manoux A. (2014). Traffic-related air pollution in relation to cognitive function in older adults. Epidemiology.

[8] Fuks K.B., Weinmayr G., Hennig F., Tzivian L., Moebus S., Jakobs H., Memmesheimer M., Kälsch H., Andrich S., Nonnemacher M. (2016). Association of long-term exposure to local industry- and traffic-specific particulate matter with arterial blood pressure and incident hypertension. Int. J. Hyg. Environ. Health.

[9] Raaschou-Nielsen O., Andersen Z.J., Jensen S.S., Ketzel M., Sorensen M., Hansen J., Loft S., Tjønneland A., Overvad K. (2012). Traffic air pollution and mortality from cardiovascular disease and all causes: A Danish cohort study. Environ. Health.

[10] Van Donkelaar A., Martin R.V., Brauer M., Kahn R., Levy R., Verduzco C., Villeneuve P.J. (2010). Global estimates of ambient fine particulate matter concentrations from satellite-based aerosol optical depth: Development and application. Environ. Health Perspect..

[11] Hoek G. (2017). Methods for assessing long-term exposures to outdoor air pollutants. Curr. Environ. Health Rep..

[12] Hoek G., Beelen R., Cd H., Vienneau D., Gulliver J., Fischer P., Briggs D. (2008). A review of land-use regression models to assess spatial variation of outdoor air pollution. Atmos. Environ..

[13] Vallero D.A. (2019). Chapter 14—Air pollution dispersion models. Air Pollution Calculations.

[14] Kloog I., Sorek-Hamer M., Lyapustin A., Coull B., Wang Y., Just A.C., Schwartz J., Broday D.M. (2015). Estimating daily PM2. 5 and PM10 across the complex geoclimate region of Israel using MAIAC satellite-based AOD data. Atmos. Environ..

[15] Prüss-Ustün A., Wolf J., Corvalán C., Bos R., Neira M., World Health Organization (WHO) (2016). Preventing Disease through Healthy Environments: A Global Assessment of the Burden of Disease from Environmental Risks.

[16] GBD 2019 Diseases and Injuries Collaborators (2020). Global burden of 369 diseases and injuries in 204 countries and territories, 1990-2019, a systematic analysis for the Global Burden of Disease Study 2019. Lancet.

[17] Akita Y., Baldasano J.M., Beelen R., Cirach M., de Hoogh K., Hoek G., Nieu-wenhuijsen M., Serre M.L., de Nazelle A. (2014). Large scale air pollution estimation method combining land use regression and chemical transport modeling in a geostatistical framework. Environ. Sci. Technol..

[18] de Hoogh K., Korek M., Vienneau D., Keuken M., Kukkonen J., Nieuwenhuijsen M.J., Badaloni C., Beelen R., Bolignano A., Cesaroni G. (2014). Comparing land use regression and dispersion modelling to assess residential exposure to ambient air pollution for epidemiological studies. Environ. Int..

[19] Wang M., Sampson P.D., Hu J., Kleeman M., Keller J.P., Olives C., Szpiro A.A., Vedal S., Kaufman J.D. (2016). Combining Land-Use Regression and Chemical Transport Modeling in a Spatiotemporal Geostatistical Model for Ozone and PM2.5. Environ. Sci. Technol..

[20] Di Q., Koutrakis P., Schwartz J. (2016). A hybrid prediction model for PM2. 5 mass and components using a chemical transport model and land use regression. Atmos. Environ..

[21] London Datastore. https://data.london.gov.uk/dataset/lsoa-atlas.

[22] London Air Quality Network (LAQN) King’s College, London. http://www.londonair.org.uk/.

[23] Air Quality England (AQE) Ricardo Energy and Environment. http://www.airqualityengland.co.uk/.

[24] Automatic Urban and Rural Network (AURN) Data Archive © Crown 2017 Copyright Defra Via https://uk-air.defra.gov.uk, Licensed under the Open Government License (OGL) v2.0. http://www.nationalarchives.gov.uk/doc/open-government-licence/version/2/.

[25] Analitis A., Barratt B., Green D., Beddows A., Samoli E., Schwartz J., Katsouyanni K. (2020). Prediction of PM_2.5_ concentrations at the locations of monitoring sites measuring PM_10_ and NOx, using generalized additive models and machine learning methods, for enhancement of the PM_2.5_ database: A case study in London. Atmos. Environ..

[26] Dimakopoulou K., Samoli E., Katsouyanni K. (2020). Spatio-temporal land use regression modelling of ozone levels in Athens, Greece. Glob. Nest J..

[27] Gryparis A., Dimakopoulou K., Pedeli X., Katsouyanni K. (2014). Spatio-temporal semiparametric models for NO2 and PM10 concentration levels in Athens, Greece. Stoten.

[28] ESRI (2011). ArcGIS Desktop: Release 10.

[29] R Core Team (2017) R: A Language and Environment for Statistical Computing. R Foundation for Statistical Computing, Vienna, Austria. https://www.R-project.org/.

[30] Wand M. SemiPar: Semiparametic Regression. R package Version 1.0-4.2 SemiPar 1.0. R Package. http://cran.r-project.org.

[31] Skamarock W.C., Klemp J.B., Dudhia J., Gill D.O., Barker D.M., Duda M.D., Huang X.-Y., Wang W., Powers J.G. (2008). A Description of the Advanced Research WRF Version 3.

[32] Byun D.W., Ching J.K.S. (1999). Science Algorithms of the EPA Models-3 Community Multiscale Air Quality (CMAQ) Modelling System.

[33] Beevers S.D., Kitwiroon N., Williams M.L., Carslaw D.C. (2012). One way coupling of CMAQ and a road source dispersion model for fine scale air pollution predictions. Atmos. Environ..

[34] CERC, ADMS Roads v4 User Guide. http://www.cerc.co.uk/environmental-software/assets/data/doc_userguides/CERC_ADMS-Roads4.0_User_Guide.pdf.

[35] Defra (2016). Emissions of Air Quality Pollutants 1990–2014. Defra, UK. https://uk-air.defra.gov.uk/assets/documents/reports/cat07/1609130906_NAEI_AQPI_Summary_Report_1990-2014_Issue1.1.pdf.

[36] Greater London Authority (GLA) (2016). The London Atmospheric Emissions Inventory 2013. https://data.london.gov.uk/dataset/london-atmospheric-emissions-inventory-2013.

[37] Beevers S.D., Westmoreland E., de Jong M.C., Williams M.L., Carslaw D.C. (2012). Trends in NOX and NO2 emissions from road traffic in Great Britain. Atmos. Environ..

[38] Vukovich Jeffrey M., Pierce T.E. The Implementation of BEIS3 within the SMOKE modeling framework. Proceedings of the 11th International Emissions Inventory Conference.

[39] Lee S., Wolberg G., Shin S. (1997). Scattered data interpolation with multilevel B-splines. IEEE Trans. Vis. Comput. Graph..

[40] Defra (2010). Evaluating the Performance of Air Quality Models. Defra, UK. https://uk-air.defra.gov.uk/assets/documents/reports/cat05/1006241607_100608_MIP_Final_Version.pdf.

[41] Danesh Yazdi M., Kuang Z., Dimakopoulou K., Barratt B., Suel E., Amini H., Lyapustin A., Katsouyanni K., Schwartz J. (2020). Predicting Fine Particulate Matter (PM2.5) in the Greater London Area: An Ensemble Approach using Machine Learning Methods. Remote Sens..

[42] Lyapustin A., Wang Y., Korkin S., Huang D. (2018). MODIS Collection 6 MAIAC algorithm. Atmos. Meas. Tech..

[43] Lin L.I.-K. (1989). A Concordance Correlation Coefficient to evaluate reproducibility. Biometrics.

[44] Bland J.M., Altman D.G. (1999). Measuring agreement in method comparison studies. Stat Methods Med. Res..

[45] Araki S., Shima M., Yamamoto K. (2018). Spatiotemporal land use random forest model for estimating metropolitan NO_2_ exposure in Japan. Sci. Total Environ..

[46] Chen J., de Hoogh K., Gulliver J., Hoffmann B., Hertel O., Ketzel M., Bauwelinck M., van Donkelaar A., Hvidtfeldt U.A., Katsouyanni K. (2019). A comparison of linear regression, regularization, and machine learning algorithms to develop Europe-wide spatial models of fine particles and nitrogen dioxide. Environ. Int..

[47] Wong P.Y., Lee H.Y., Chen Y.C., Zeng Y.T., Chern Y.R., Chen N.T., Candice Lung C.S., Su H.J., Wu C.D. (2021). Using a land use regression model with machine learning to estimate ground level PM2.5. Environ. Pollut..

[48] de Hoogh K., Chen J., Gulliver J., Hoffmann B., Hertel O., Ketzel M., Bauwelinck M., van Donkelaar A., Hvidtfeldt U.A., Katsouyanni K. (2018). Spatial PM2.5, No2, O3 and bc models for western Europe–evaluation of spatiotemporal stability. Environ. Int..

[49] Chen J., de Hoogh K., Gulliver J., Hoffmann B., Hertel O., Ketzel M., Weinmayr G., Bauwelinck M., van Donkelaar A., Hvidtfeldt U.A. (2020). Development of Europe-wide models for particle elemental composition using supervised linear regression and random forest. Environ. Sci. Technol..

[50] Tripathy S., Tunno B.J., Michanowicz D.R., Kinnee E., Shmool J.L., Gillooly S., Clougherty J.E. (2019). Hybrid land use regression modeling for estimating spatio-temporal exposures to PM_2.5_, BC, and metal components across a metropolitan area of complex terrain and industrial sources Sci. Total Environ..

[51] Szpiro A.A., Sheppard L., Lumley T. (2011). Efficient measurement error correction with spatially misaligned data. Biostatistics.

[52] Samoli E., Butland B.K., Rodopoulou S., Atkinson R.W., Barratt B., Beevers S.D., Beddows A., Dimakopoulou K., Schwartz J.D., Yazdi M.D. (2020). The impact of measurement error in modeled ambient particles exposures on health effect estimates in multilevel analysis. Environ. Epidemiol..

[53] Butland B.K., Samoli E., Atkinson R.W., Barratt B., Beevers S.D., Kitwiroon N., Dimakopoulou K., Rodopoulou S., Schwartz J.D., Katsouyanni K. (2020). Comparing the performance of air pollution models for nitrogen dioxide and ozone in the context of a multilevel epidemiological analysis. Environ. Epidemiol..

